# The Application of Fat-Free Mass Index for Survival Prediction in Cancer Patients With Normal and High Body Mass Index

**DOI:** 10.3389/fnut.2021.714051

**Published:** 2021-08-04

**Authors:** Xi Zhang, Qi Zhang, Li-jin Feng, Kang-Ping Zhang, Meng Tang, Meng-meng Song, Guo-tian Ruan, Xiao-wei Zhang, Wei Li, Fu-xiang Zhou, Ming-Hua Cong, Han-Ping Shi

**Affiliations:** ^1^Department of Gastrointestinal Surgery, Clinical Nutrition, Beijing Shijitan Hospital, Capital Medical University, Beijing, China; ^2^Department of Radiotherapy, Affiliated Hospital of Hebei University, Baoding, China; ^3^Beijing International Science and Technology Cooperation Base for Cancer Metabolism and Nutrition, Beijing, China; ^4^Department of Pathology, Shanghai Tenth People's Hospital, Tongji University, School of Medicine, Shanghai, China; ^5^Cancer Center of the First Hospital of Jilin University, Changchun, China; ^6^Department of Oncology, Zhongnan Hospital of Wuhan University, Wuhan University, Wuhan, China; ^7^Department of Comprehensive Oncology, Cancer Hospital, Chinese Academy of Medical Sciences, Beijing, China

**Keywords:** FFMI, normal/high BMI, survival, prognosis, cancer patients

## Abstract

**Background:** Fat-free mass (FFM) depletion can be masked by a stable body weight or weight gain in the presence of a normal or high body mass index (BMI). This study investigated the prognostic value of low fat-free mass index (FFMI) in cancer patients with normal or high BMI.

**Methods:** This multicenter retrospective cohort study included 1,602 cancer patients with normal/high BMI. The association of FFMI with patients' overall survival (OS) was analyzed by the Kaplan-Meier method and a Cox model.

**Results:** In this analysis, there were 974 (60.8%) females and 628 (39.2%) males. Low FFMI was associated with worse OS when compared with those patients with normal FFMI. After multivariate adjustment, low FFMI was demonstrated to be an independent unfavorable prognostic factor (HR: 1.69; 95% CI: 1.28, 2.23; *P* < 0.001) in cancer patients with normal/high BMI. For specific tumor type, low FFMI was found to be associated with worse prognosis in patients with lung cancer, breast cancer and upper gastrointestinal cancer. In subgroup analysis, the association of low FFMI with worse survival was significantly modified by weight loss (*P* for interaction = 0.012), and those patients with concurrent low FFMI and weight loss showed the worst prognosis (HR: 3.53; 95% CI: 2.04, 6.11; *P* < 0.001).

**Conclusion:** Low FFMI was associated with worse prognosis in cancer patients with normal/high BMI. This study highlights the usefulness of FFMI for prognostic estimation in these patients.

## Introduction

Cancer has been associated with fat-free mass (FFM) loss, mainly the muscle mass depletion, owing to reduced food intake, elevated energy expenditure, and excess catabolism and inflammation (1). The resulting increased muscle protein breakdown leads to a loss of muscle function and depletion of protein reserves (2). FFM loss has also been proposed to be a predictor of severe toxicity following cancer treatment (3), negative efficacy of treatment (4) and poorer survival (5). However, the simple measure of BMI or percentage of weight loss does not distinguish between the deterioration of fat and muscle mass (6). The fat-free mass index (FFMI) can be easily measured by bioelectrical impedance analysis (BIA), and it can provide far more valuable information than BMI from both functional and metabolic points of view (7, 8). Thus, the Global Leadership Initiative on Malnutrition (GLIM) recommended low FFMI for the muscle mass evaluation and malnutrition diagnosis (9). Previous studies have demonstrated that underweight BMI patients have a greater risk of morbidity and mortality than normal/high BMI patients (10, 11), and this outcome is plausible because low BMI categories include more patients with lower physiologic resilience and metabolic reserves, which are needed to withstand the catabolic burden of the tumor and the treatment process. With the growing overweight and obesity epidemic, a number of cancer patients were in the normal or even high BMI range even though they may have lost a considerable and clinically relevant amount of muscle mass (12). However, the prognostic information of low FFMI is lacking for these patients. Therefore, this large-scale retrospective cohort study was conducted to explore the effect of low FFMI on prognosis estimation in cancer patients with normal or high BMI.

## Materials and Methods

### Study Design and Population

A multicenter, retrospective study was conducted in cancer patients with normal/high BMI between November 2013 and August 2018. All of the patients were admitted for cancer treatments, including surgery, chemotherapy, radiotherapy and other anti-cancer therapies. If patients experienced multiple hospitalizations, only the data for the first admission were analyzed. The inclusion criteria were: (1) age >18 years old; (2) length of hospital stay longer than 48 h; (3) diagnosis of solid tumors at any stage; (4) patients presented with a BMI ≥18.5 kg/m^2^ if <70 years old and ≥20.0 kg/m^2^ if >70 years old. We excluded patients if they had incomplete clinical data, lacked follow-up data or reported edema or amputations. In addition, we further excluded participants with BMI >36 kg/m^2^, since BIA might be inaccurate in severely obese subjects (13). Ethical approval was obtained from the participating institutions through their respective institutional review boards.

### Patient Characteristics

The clinicopathologic variables included age, gender, primary tumor site, pathologic stage, Karnofsky Performance Status (KPS), alcohol consumption, smoking status, quality of life, Nutritional RiskScreening-2002 (NRS-2002) score, hemoglobin, albumin, European Organization for Research and Treatment of Cancer Quality of Life Questionnaire (EORTC QLQ-C30) summary score, neutrophil-to-lymphocyte ratio (NLR), platelet-to-lymphocyte ratio (PLR) and previous treatments (surgery, chemotherapy and radiotherapy). Normal (18.5–23.9 kg/m^2^), overweight (24.0–27.9 kg/m^2^) and obesity (≥28.0 kg/m^2^) BMI ranges were defined according to a reclassification of BMI for Chinese adults released by the Ministry of Health of the People's Republic of China (14). The tumor (T), node (N) and metastasis (M) categories were not included in this analysis since each tumor type has distinct T/N/M categories. The KPS data were converted to the Eastern Cooperative Oncology Group performance status (ECOG PS) using the following categories: KPS 100 (ECOG PS 0), KPS 90 to 80 (ECOG PS 1), KPS 70 to 60 (ECOG PS 2), KPS 50 to 40 (ECOG PS 3), and KPS 30 to 0 (ECOG PS 4) (15). The QLQ-C30 summary score is calculated as the mean of the combined 13 QLQ-C30 scale and item scores (excluding global QoL and financial impact), with a higher score indicating a better QoL (16, 17). All pathological staging was defined according to the 8th edition of the AJCC TNM staging system.

### Body Composition and Anthropometric Measurements

FFM was evaluated by BIA using the InBody S10 (Beijing, China) body composition analyzer. The analysis was conducted with the patients in the supine position, with two electrodes for each foot and hand attached at the four extremities. All of the procedures were conducted according to recommendations from the manufacturers (18). BMI was calculated as body weight (a weighing scale adjusted to 0.1 kg) divided by the square of height. FFM can be divided by height squared to be converted into the FFM index (FFMI). Moreover, anthropometric data such as the mid-arm muscle and calf circumferences were also examined. The knee was flexed to 90 degrees with the feet and ankles relaxed, and the largest calf circumference (CC) was measured using a standard tape measure with a 0.1-cm increment. The mid-arm circumference (MAC) and triceps skinfold (TSF) were measured in 0.1- and 1-mm increments, respectively, at the midpoint between the acromion and the olecranon. The MAC was measured using a plastic metric tape, and the TSF was measured using skinfold calipers. The mid-arm muscle circumference (MAMC) was obtained using the following formula: MAMC (mm) = mid-arm circumference (mm)–[3.14 × triceps skinfold(mm)]. Hand grip strength (HGS) was also measured from the patient's dominant hand with a Jamar dynamometer. The patients were asked to recall what their weight was 6 months prior, and this was compared to the weight measured at the time of admission.

### Statistical Analysis

All of the data are expressed as the mean ± standard deviation, median (interquartile range, IQR), or absolute number and proportion as appropriate. Comparison of continuous variables was performed using Student's independent *t*-test, or the Mann-Whitney test for data without a normal distribution. Categorical variables were compared using the χ^2^-test or Fisher's exact test. The optimal cut-off values of FFMI, NLR, and PLR for survival prediction were determined with the aid of maximally selected rank statistics (19). Multivariate analysis was performed by Cox regression analysis using backward selection to identify the independent significance of different parameters. Two sensitivity analyses were performed as follows: one analysis excluded patients who died within 6 months to reduce the potential impact of reverse causation. Besides, propensity score-matching was used to control of selection bias and potential confounding (20). The propensity scores were estimated using a logistic regression model according to the following variables: age, gender, ECOG PS, pathological stage, weight loss, tumor types, radiotherapy, reduced food intake and NLR. Propensity score-matching was performed with a caliper width of 0.1 multiplied by the standard deviation for the linearly transformed propensity scores. After amending these confounding factors, we reevaluated the prognostic significance of FFMI in cancer patients with normal and high body mass index. *P* < 0.05 was considered statistically significant. All of the analyses were performed using R software, version 3.6.1.

## Results

### Characteristics of Patients

Initially, 1,812 cancer patients with normal or high BMI were enrolled in the study. Two hundred ten patients were excluded because of missing data for one or more of the variables used in our analysis. Consequently, 1,602 patients were eligible for the final analyses (Supplementary Figure 1). Table 1 summarizes the baseline characteristics of the patients. The mean age was 56.65 years old, and 39.2% were male. The patients had a mean BMI of 24.13 kg/m^2^. The median follow-up time was 29.95 months, and 373 patients died during follow-up.

**Table 1:** Detailed baseline characteristics of the study population.

**Characteristics**	**Normal FFMI**	**Low FFMI**	***P*-value**
	***n* = 1,432**	***n* = 170**	
Age, years, n (%)			0.252
≤ 65	1,172 (81.8%)	133 (78.2%)	
>65	260 (18.2%)	37 (21.8%)	
Gender, n (%)			0.545
Male	565 (39.5%)	63 (37.0%)	
Female	867 (60.5%)	107 (63.0%)	
ECOG performance status, n (%)			0.064
≤ 1	1,040 (72.6%)	112 (65.9%)	
>1	392 (27.4%)	58 (34.1%)	
Smoking, n (%)			0.787
Absent	903 (63.1%)	109 (64.1%)	
Present	529 (36.9%)	61 (35.9%)	
Drinking, n (%)			0.906
Absent	1,174 (82.0%)	140 (82.4%)	
Present	258 (18.0%)	30 (17.6%)	
TNM stages, n (%)			0.140
I	299 (20.9%)	28 (16.5%)	
II	370 (25.8%)	50 (29.5%)	
III	456 (31.8%)	46 (27.0%)	
IV	307 (21.5%)	46 (27.0%)	
M category, n (%)			0.085
M0	1,135 (79.3%)	125 (73.5%)	
M1	297 (20.7%)	45 (26.5%)	
BMI category, n (%)			<0.001
Normal	676 (47.2%)	154 (90.6%)	
Overweight	576 (40.2%)	14 (8.2%)	
Obese	180 (12.6%)	2 (1.2%)	
NRS-score, n (%)			0.001
NRS ≥ 3	326 (22.8%)	59 (34.7%)	
NRS <3	1,106 (77.2%)	111 (65.3%)	
Weight-loss, n (%)			<0.001
Absent	1,242 (86.7%)	127 (74.7%)	
Present	190 (13.3%)	43 (25.3%)	
Reduced food intake			0.025
Absent	1,074 (75.0%)	114 (67.1%)	
Present	358 (25.0%)	56 (32.9%)	
Tumor types, n (%)			0.022
Lung cancer	499 (34.8%)	49 (28.8%)	
UGIC	90 (6.3%)	21 (12.4%)	
CRC	174 (12.2%)	27 (15.9%)	
Breast cancer	434 (30.3%)	52 (30.6%)	
Other cancer	235 (16.4%)	21 (12.3%)	
Previous treatments, n (%)			
Surgery	883 (61.7%)	112 (65.9%)	0.284
Chemotherapy	978 (68.3%)	109 (64.1%)	0.270
Radiotherapy	184 (12.8%)	35 (20.6%)	0.005
QLQ-C30, median (range)			
Summary score	88.95 (13.84)	87.37 (18.40)	0.018
BMI, kg/m^2^, mean ± SD	24.49 ± 3.04	21.15 ± 2.03	<0.001
MAMC, cm, mean ± SD	21.87 ± 2.72	20.41 ± 2.40	<0.001
MAC, cm, mean ± SD	27.85 ± 3.11	25.49 ± 2.64	<0.001
TSF, mm, mean ± SD	19.05 ± 6.05	16.18 ± 5.24	<0.001
HGS, kg, mean ± SD	25.19 ± 9.19	21.71 ± 8.03	<0.001
CC, cm, mean ± SD	35.04 ± 3.59	32.66 ± 3.40	<0.001
Albumin, g/L, mean ± SD	39.62 ± 4.86	39.35 ± 5.43	0.539
Hemoglobin, g/L,mean ± SD	126.11 ± 18.49	122.53 ± 18.35	0.017
NLR, median (range)	2.27 (2.09)	2.48 (2.74)	0.017
PLR, median (range)	143.3 (100.6)	160.6 (118.5)	0.001

*ECOG, Eastern Cooperative Oncology Group; UGIC, upper gastrointestinal cancer; CRC, colorectal cancer; BMI, body mass index; M, metastasis; NRS-2002, Nutritional Risk Screening-2002; FFMI, fat free mass index; TSF, triceps skinfold; MAC, Mid-arm circumference; MAMC, Mid-arm muscular circumference; CC, calf-circumference; HGS, hand grip strength; NLR, neutrophil-to-lymphocyte ratio; PLR, platelet-to-lymphocyte ratio; SD, standard deviation*.

### Association of the FFMI With Clinicopathologic Variables

**Figure 1:**
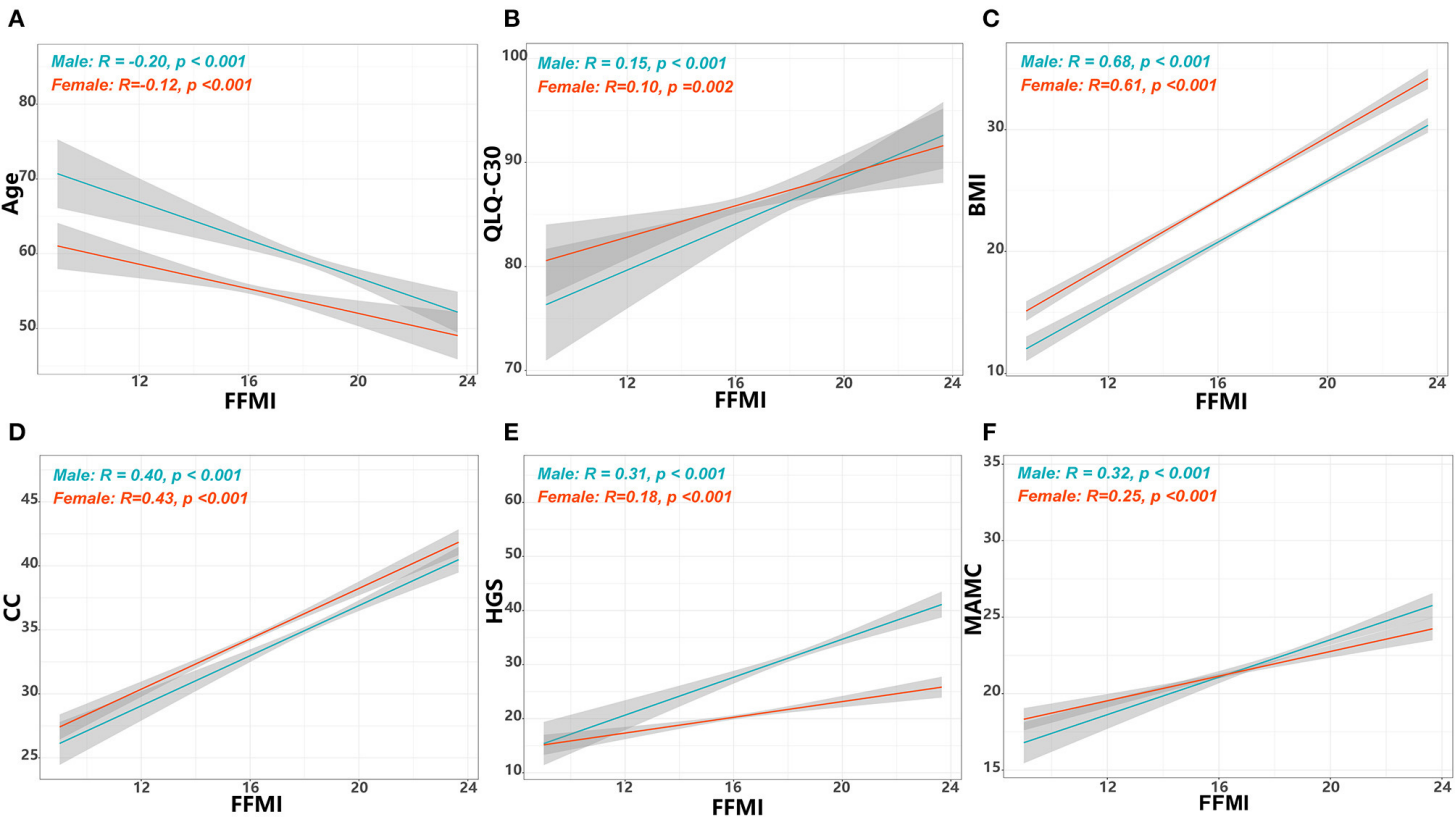
Correlations of FFMI with age, summary score of QLQ-C30 and anthropometric measurements. The correlations of FFMI with age **(A)**, summary score of QLQ-C30 **(B)**, BMI **(C)**, CC **(D)**, **(E)** HGS, and MAMC **(F)**. FFMI, fat free mass index; BMI, body mass index; CC, calf circumference; MAMC, mid-arm muscle circumference; HGS, hand grip strength; QLQ-C30, Quality of Life Questionnaire.

FFMI showed moderate positive correlations with BMI and CC, weak positive correlations with QLQ-C30 summary score, MAMC and HGS, but showed weak negative correlations with age (Figure 1). The optimal thresholds of the FFMI were 16.3 for males and 14.5 for females (Supplementary Figure 2). Patients with low FFMI tended to present with poorer ECOG performance and QLQ-C30 summary score, high NRS-score, metastatic tumors, low hemoglobin, high NLR and

PLR, receiving radiotherapy, and upper gastrointestinal cancer (UGIC). In addition, weight loss, reduced food intake, low HGS, low MAMC, low CC and low TSF were more frequently seen in patients with low FFMI (Table 1).

### Association of the FFMI With Overall Survival

**Figure 2:**
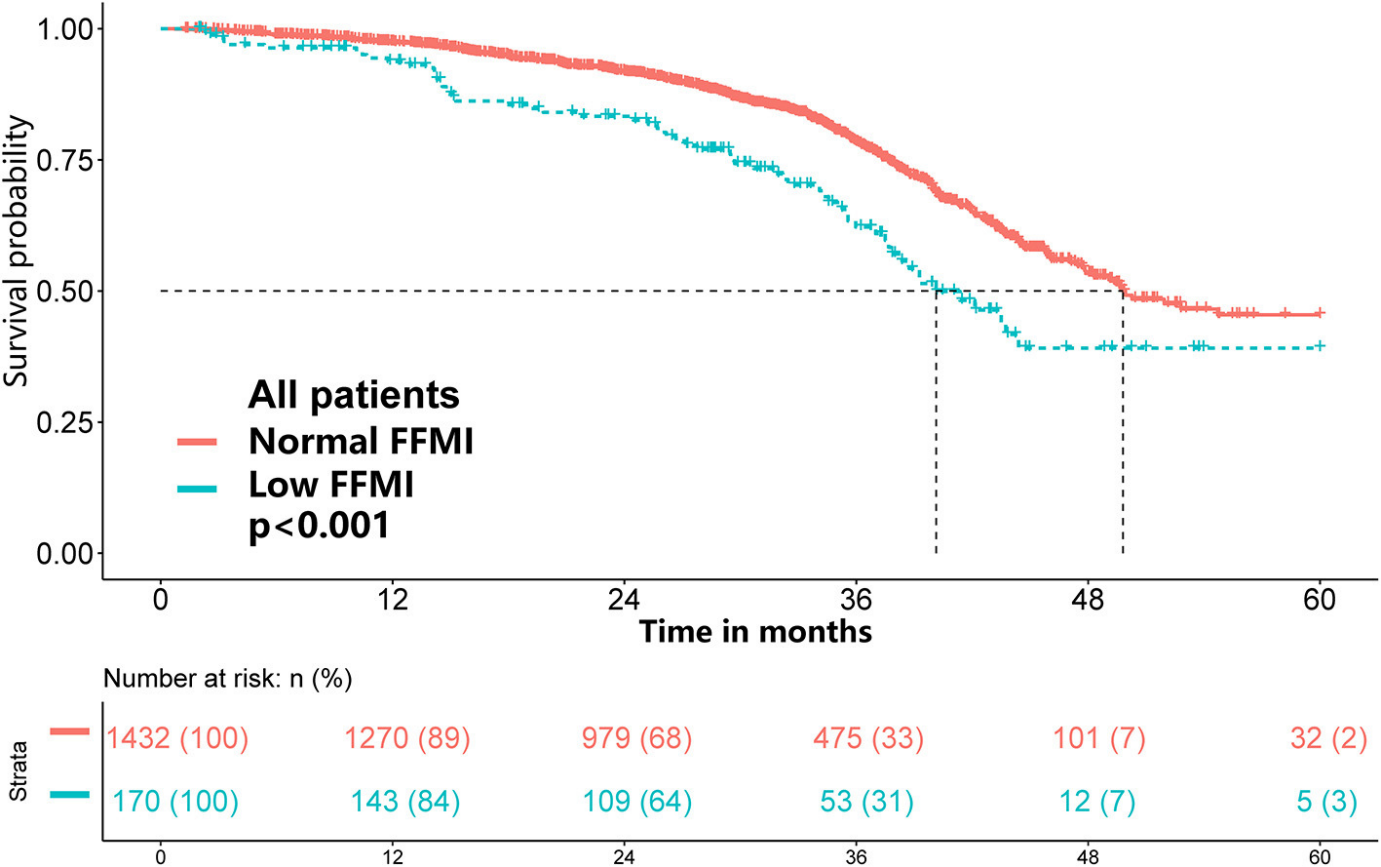
Results of the Kaplan-Meier survival analysis stratified by FFMI in cancer patients with normal/high BMI.

**Figure 3:**
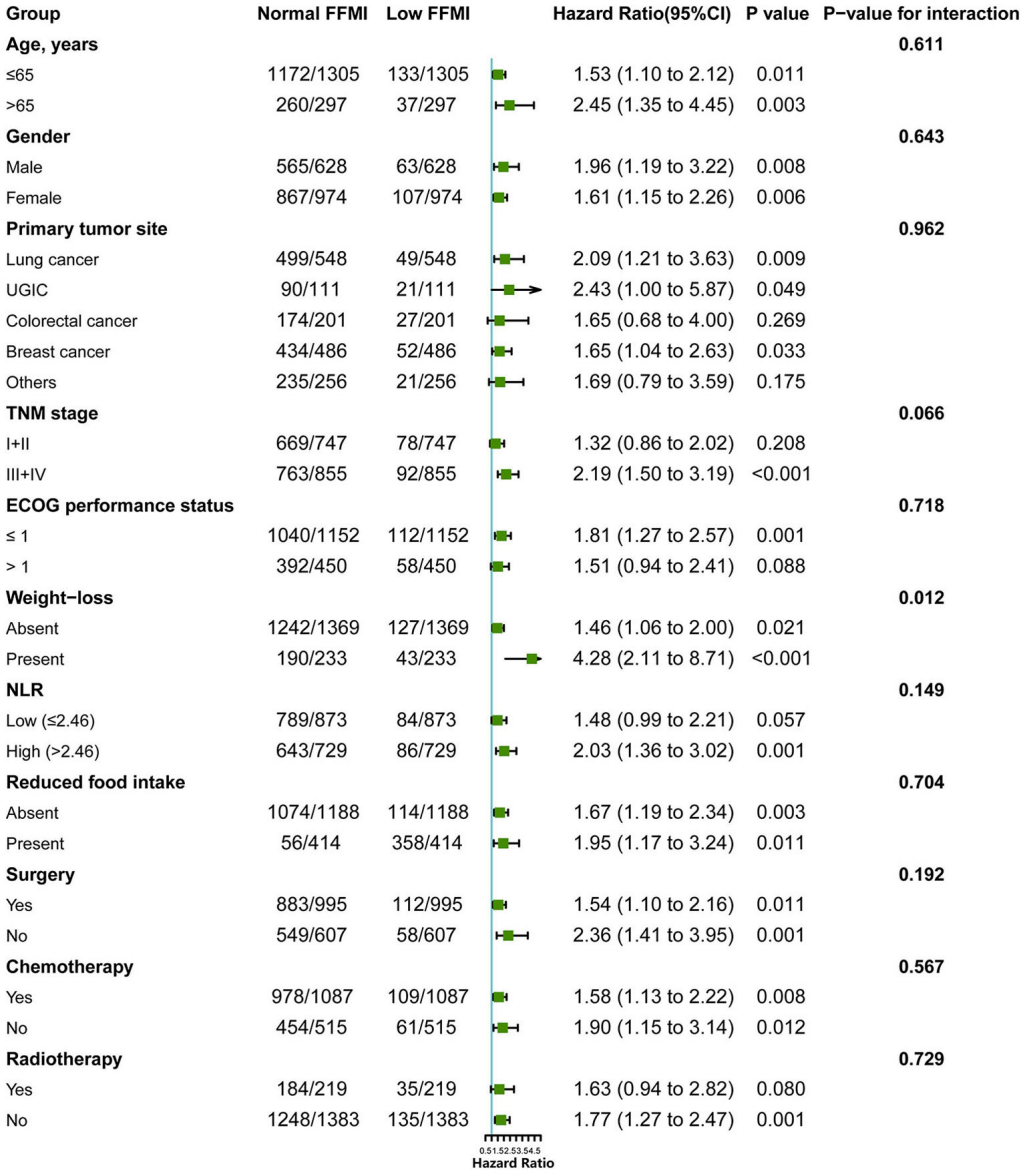
Subgroup analysis for evaluating the prognostic effect of FFMI on OS in cancer patients with normal/high BMI. FFMI, fat free mass index; UGIC, upper gastrointestinal cancer; ECOG, Eastern Cooperative Oncology Group; NLR, neutrophil-to-lymphocyte ratio. Adjusted for tumor type, ECOG performance status, TNM stage, reduced food intake and radiotherapy.

Of the 1,602 eligible patients, 10.6% had a low FFMI. Low FFMI was associated with shorter OS than normal FFMI (Figure 2). Multivariate analysis identified low FFMI as an unfavorable prognostic factor for OS (HR: 1.69; 95% CI: 1.28, 2.23; *P* < 0.001) after adjustment for TNM stage, tumor type, ECOG performance status, NLR, weight loss, reduced food intake and radiotherapy (Table 2). Low FFMI was also confirmed as an independent prognostic factor using the sensitivity analysis by excluding patients who died within 6 months or the propensity score-matching analysis (Supplementary Figure 3 and Supplementary Table 1). In addition, low FFMI was significantly associated with poorer OS in patients with lung cancer and breast cancer, and tended to be associated with shorter OS in patients with UGIC (Supplementary Figure 4). After multivariate adjustment, low FFMI was an independent prognostic factor for OS in patients with lung cancer, breast cancer and UGIC, but not for these patients with colorectal cancer (Figure 3). Furthermore, the Kaplan-Meier analysis showed that low FFMI was significantly associated with worse OS in patients received surgery and chemotherapy, and was likely to be associated with shorter OS in patients received radiotherapy (Supplementary Figure 5). Multivariate analysis confirmed low FFMI as an independent worse prognostic factor for OS in patients received surgery and chemotherapy (Figure 3).

**Table 2:** Univariate and multivariate Cox regression analysis of OS in cancer patients with normal/high BMI.

**Variables**	**No. of patients (%)**	**Univariate Analysis**			**Multivariate Analysis**		
		**Hazard Ratio**	**95% CI**	***P-value***	**Hazard Ratio**	**95% CI**	***P-value***
Age, years							
≤ 65	1,305	Reference					
>65	297	0.91	0.68 to 1.21	0.496			
Gender							
Male	628	1.06	0.85 to 1.32	0.606			
Female	974	Reference					
Primary tumor site							
Lung cancer	548	Reference			Reference		
UGIC	111	1.00	0.66 to 1.53	0.995	0.88	0.57–1.38	0.587
CRC	201	0.54	0.37 to 0.78	0.001	0.53	0.36–0.77	<0.001
Breast cancer	486	0.83	0.64–1.06	0.138	0.97	0.73–1.29	0.830
Other	256	0.97	0.71–1.33	0.854	1.07	0.76–1.51	0.696
Stages							
I	327	Reference			Reference		
II	420	1.16	0.86–1.55	0.326	1.20	0.89–1.62	0.217
III	502	1.27	0.95–1.70	0.100	1.40	1.03–1.90	0.033
IV	353	1.64	1.20–2.26	0.002	1.58	1.11–2.25	0.011
M category							
M0	1,259	Reference					
M1	343	1.42	1.10–1.84	0.007			
ECOG performance status							
≤ 1	1,152	Reference			Reference		
>1	450	1.57	1.27–1.95	<0.001	1.33	1.06–1.67	0.012
Weight-loss							
Absent	1,369	Reference					
Present	233	1.36	1.02–1.80	0.034			
NRS-score							
NRS <3	1,217	Reference					
NRS ≥ 3	385	1.27	1.00–1.61	0.048			
FFMI							
Normal	1,432	Reference			Reference		
Low	170	1.75	1.33–2.30	<0.001	1.69	1.28–2.23	<0.001
HGS							
Normal	1,297	Reference					
Low	305	1.20	0.94–1.54	0.149			
Reduced food intake							
Absent	1,188	Reference			Reference		
Present	414	1.64	1.32–2.05	<0.001	1.48	1.17–1.89	0.001
Surgery							
No	607	Reference					
Yes	995	1.02	0.82–1.29	0.806			
Chemotherapy							
No	515	Reference					
Yes	1,087	1.03	0.82–1.29	0.780			
Radiotherapy							
No	1,383	Reference			Reference		
Yes	219	1.53	1.19–1.96	0.001	1.48	1.14–1.90	0.003
Serum albumin, g/L							
Normal (>35)	1,310	Reference					
Abnormal (≤ 35)	292	0.95	0.71–1.26	0.713			
NLR							
Low (≤ 2.46)	873	Reference					
High (>2.46)	729	1.27	1.03–1.56	0.024			
PLR							
Low (≤ 166.9)	969	Reference					
High (>166.9)	633	1.20	0.98–1.48	0.079			
Hemoglobin, g/dL							
Normal (≥12)	1,058	Reference					
Abnormal (<12)	544	1.16	0.94–1.44	0.165			

*UGIC, upper gastrointestinal cancer; CRC, colorectal cancer; M, metastasis; ECOG, Eastern Cooperative Oncology Group; M, metastasis; FFMI, fat free mass index; NLR, neutrophil-to-lymphocyte ratio; PLR, platelet-to-lymphocyte ratio*.

### Stratified Analyses by Potential Modifiers

**Figure 4:**
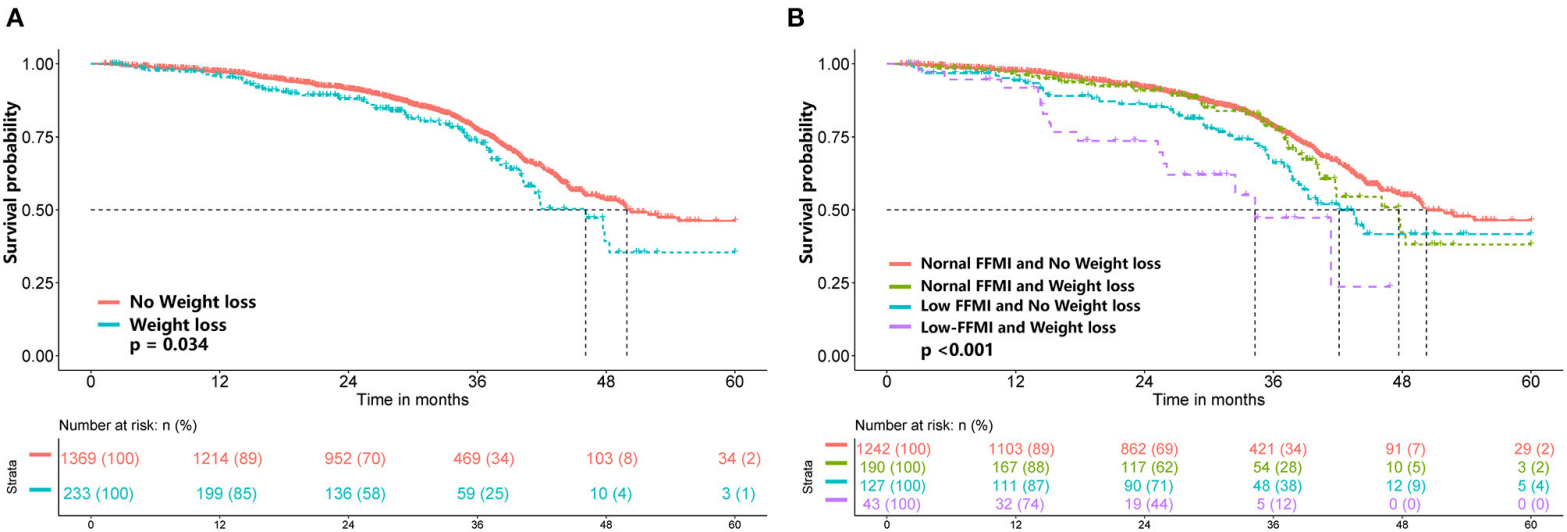
Kaplan-Meier survival analysis of weight loss **(A)** and FFMI combined with weight loss **(B)**. FFMI, fat free mass index.

To further elucidate the potential effect of FFMI on prognosis, stratified analyses were performed in several subgroups. The association of low FFMI with worse survival was significantly modified by weight loss (*P* for interaction = 0.012), but not by other potential modifiers including age (*P* for interaction = 0.611), gender (*P* for interaction = 0.643), primary tumor site (*P* for interaction = 0.962), TNM stage (*P* for interaction = 0.066), ECOG performance status (*P* for interaction = 0.718), NLR (*P* for interaction = 0.149), reduced food intake (*P* for interaction = 0.704), surgery (*P* for interaction = 0.192), chemotherapy (*P* for interaction = 0.567) and radiotherapy (*P* for interaction = 0.729). When combined with weight loss, low FFMI-weight loss was associated with the worst OS. Intriguingly, weight loss may not confer the poorer prognosis when FFMI was normal (Figure 4). Multivariate analysis, including age, gender, TNM stage, tumor type, ECOG, NLR, weight loss, reduced food intake and radiotherapy, identified the combined presence of low FFMI with weight loss (HR: 3.53; 95% CI:2.04, 6.11; *p* < 0.001) and low FFMI without weight loss (HR: 1.45; 95% CI:1.05, 1.99; *p* = 0.022) as significant worse prognostic factors for OS (Supplementary Table 2).

## Discussion

This study was a large-scale study to examine the relationship between low FFMI and mortality in cancer patients with normal or high BMI. Although the GLIM criteria recommended cut-off values for a low FFMI (FFMI <15 kg/m^2^ in women and <17 kg/m^2^ in men) based on Swiss reference material (9), it might be reasonable that Asians are more likely to have lower FFMI values than Westerns. Moreover, cut-off values of low FFMI need to be linked to the fact that female usually have a lower FFMI and higher FMI than male. Therefore, the current study calculated sex-specific cut-off points of FFMI as 14.5 kg/m^2^ for females and 16.3 kg/m^2^ for males. Using the cut-off points, low FFMI was found in 10.6% of cancer patients with normal/high BMI, which is slightly higher than the percentage reported in previous study. Willemsen et al. (21) found that the incidence of low FFMI was 8.7% in locally advanced head and neck squamous cell carcinoma patients (LAHNSCC) with normal BMI (≥21 kg/m^2^). In addition, the results of the present study showed that low FFMI was an independent prognostic factor in the settings of lung cancer, breast cancer and UGIC. In line with our result, Burtin et al. (22) found low FFMI was associated with worse prognosis in non-small cell lung cancer patients with good performance status (WHO PS 0 or 1). Song et al. (23) demonstrated higher skeletal muscle volume was associated with more favorable prognosis than those with lower muscle volume in patients with breast cancer. Furthermore, we found low FFMI independently associated with worse prognosis in cancer patients received surgery and chemotherapy, but tended to be a worse prognostic factor for patients treated with radiotherapy. One potential reason might be the limited number of patients with low FFMI in radiotherapy group. A recent study found low FFMI was an unfavorable prognostic factor for OS in LAHNSCC patients undergoing chemoradiation or bioradiation treatment (21).

Muscle mass reduction is driven by a variable combination of decreased calorie/protein intake, metabolic changes and inflammation (1). This fact is also partly reflected in the current study that a low FFMI was more frequently seen in patients at risk of malnutrition (NRS score ≥3) and in those with high levels of inflammatory biomarkers (high NLR and PLR). Moreover, muscle loss could be linked to a poor prognosis not only pathophysiologically but also indirectly by reducing daily activities (24). The current study found patients with low FFMI usually had poorer ECOG performance and summary score of QLQ-C30. Furthermore, a recent study reported a significant association between low FFMI and accelerated hospitalization in colorectal cancer patients (25). One explanation of this phenomenon is that patients with low FFMI have decreased metabolically active body cell mass, which is needed to withstand operative stress and complication development.

The present study found that weight loss combined with low FFMI was associated with worse OS in cancer patients with normal/high BMI. However, there was no significant difference in survival between patients with and without weight loss in the subgroup with normal FFMI, indicating that the FFMI might well represent the association of weight loss and survival. Similarly, a prospective cohort study found that critical weight loss patients with low FFMI had a higher mortality risk, but critical weight loss and normal FFMI patients did not (26). These findings suggest that weight loss only becomes relevant when protein reserves are depleted. In addition, advanced stages of cancer are often associated with altered energy expenditure and lower dietary intake, which can affect body composition. In the present study, the prognostic value of a low FFMI tended to be more pronounced in patients with advanced TNM stages (III and IV). A recent systematic review also reported that low muscle mass was associated with poorer OS in patients with incurable cancer (27). These results emphasize the significance of assessing the FFMI in these patients with advanced TNM stages. In addition, evidence suggests that muscle loss, when combined with increased systemic inflammatory response, worsens the clinical outcomes of cancer patients (28–30). In the present study, we found that the association between low FFMI and poorer prognosis tended to be modified by NLR; low FFMI exhibited more pronounced prognostic significance in patients with high NLR.

Some limitations are associated with the current study. First, FFMI data were retrospectively collected, and were not available for some patients, therefore, the results of this study might be subject to selection bias. However, the relatively large sample size might partially compensate for this limitation. Second, computed tomography (CT) is considered the gold standard in measuring body composition, but was not available in the present cohort. However, BIA measurement is a feasible, non-invasive measure with low cost, and could become widely available in clinical research settings. Finally, due to the limited number of low FFMI cases in overweight and obese BMI subsetting, subgroup analysis could not be conducted in these patients.

In conclusion, this study found low FFMI was associated with worse prognosis in cancer patients with normal/high BMI. The influence of low FFMI on worse prognosis is likely to be more pronounced in patients with weight loss. This study highlights the usefulness of FFMI for prognostic estimation in cancer patients with normal/high BMI.

## Data Availability Statement

The raw data supporting the conclusions of this article will be made available by the authors, without undue reservation.

## Ethics Statement

The studies involving human participants were reviewed and approved by This study was approved by the Medical Ethics Committee of First Affiliated Hospital of Sun yat-sen University, and registered in the Chinese Clinical Trial Registry (NO ChiCTR1800020329, http://www.chictr.org.cn/). Written informed consent for participation was not required for this study in accordance with the national legislation and the institutional requirements.

## Author Contributions

H-PS: conceptualization and methodology. XZ: data curation and writing the original draft preparation. QZ: visualization, investigation, and data curation. L-jF: methodology and investigation. K-PZ: software. MT: validation and visualization. M-mS, G-tR, and X-wZ: writing–reviewing and editing. WL, F-xZ, and M-HC: supervision and investigation. All authors contributed to the article and approved the submitted version.

## Conflict of Interest

The authors declare that the research was conducted in the absence of any commercial or financial relationships that could be construed as a potential conflict of interest.

## Publisher's Note

All claims expressed in this article are solely those of the authors and do not necessarily represent those of their affiliated organizations, or those of the publisher, the editors and the reviewers. Any product that may be evaluated in this article, or claim that may be made by its manufacturer, is not guaranteed or endorsed by the publisher.
