# Supplementary material for: The Application of Fat-Free Mass Index for Survival Prediction in Cancer Patients With Normal and High Body Mass Index
Source: Front Nutr. 2021 Aug 4;8:714051. doi: 10.3389/fnut.2021.714051 (PMC8371389; doi:10.3389/fnut.2021.714051)
Supplement: Supplementary file 1 [file Data_Sheet_1.docx]

**Figure S1** Flow chart of enrolled cancer patients.


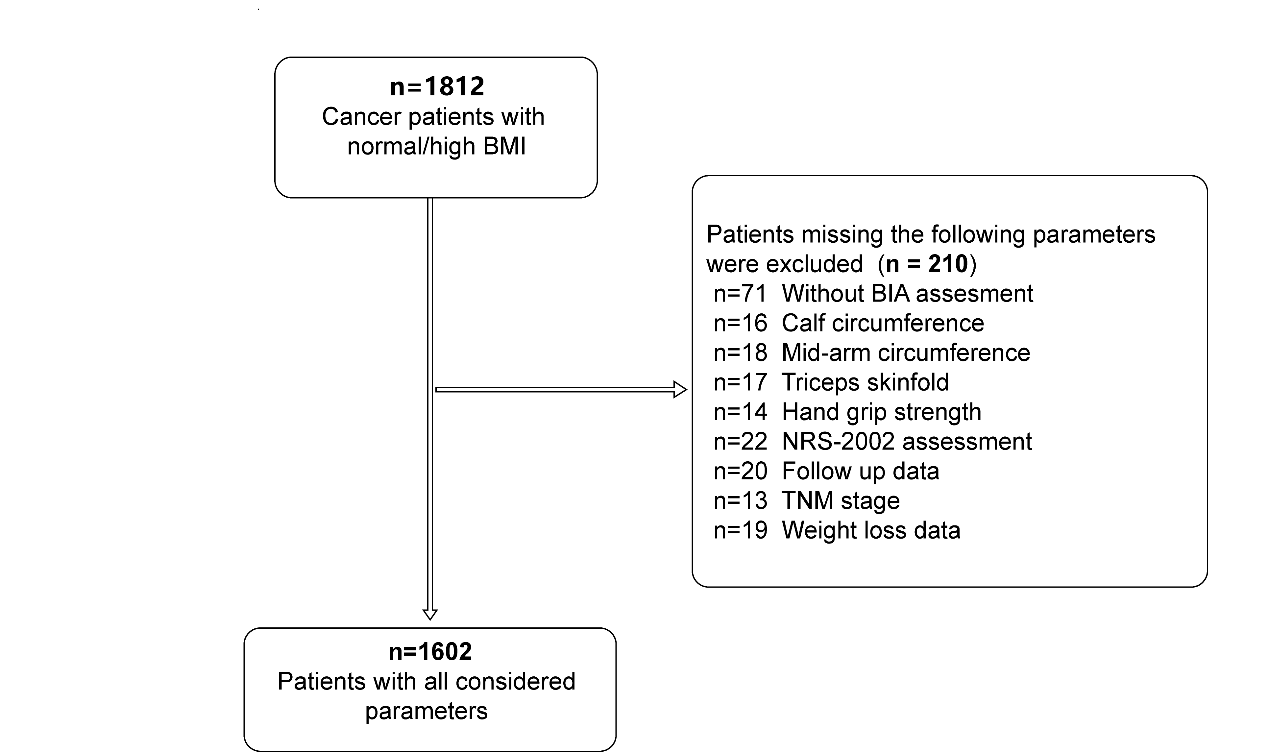


BMI; body mass index; NRS-2002, Nutritional Risk Screening-2002; BIA, bioelectrical impedance analysis.

**Figure S2** Determining cut-off values of low FFMI based on sex-specific (male, female) strata.
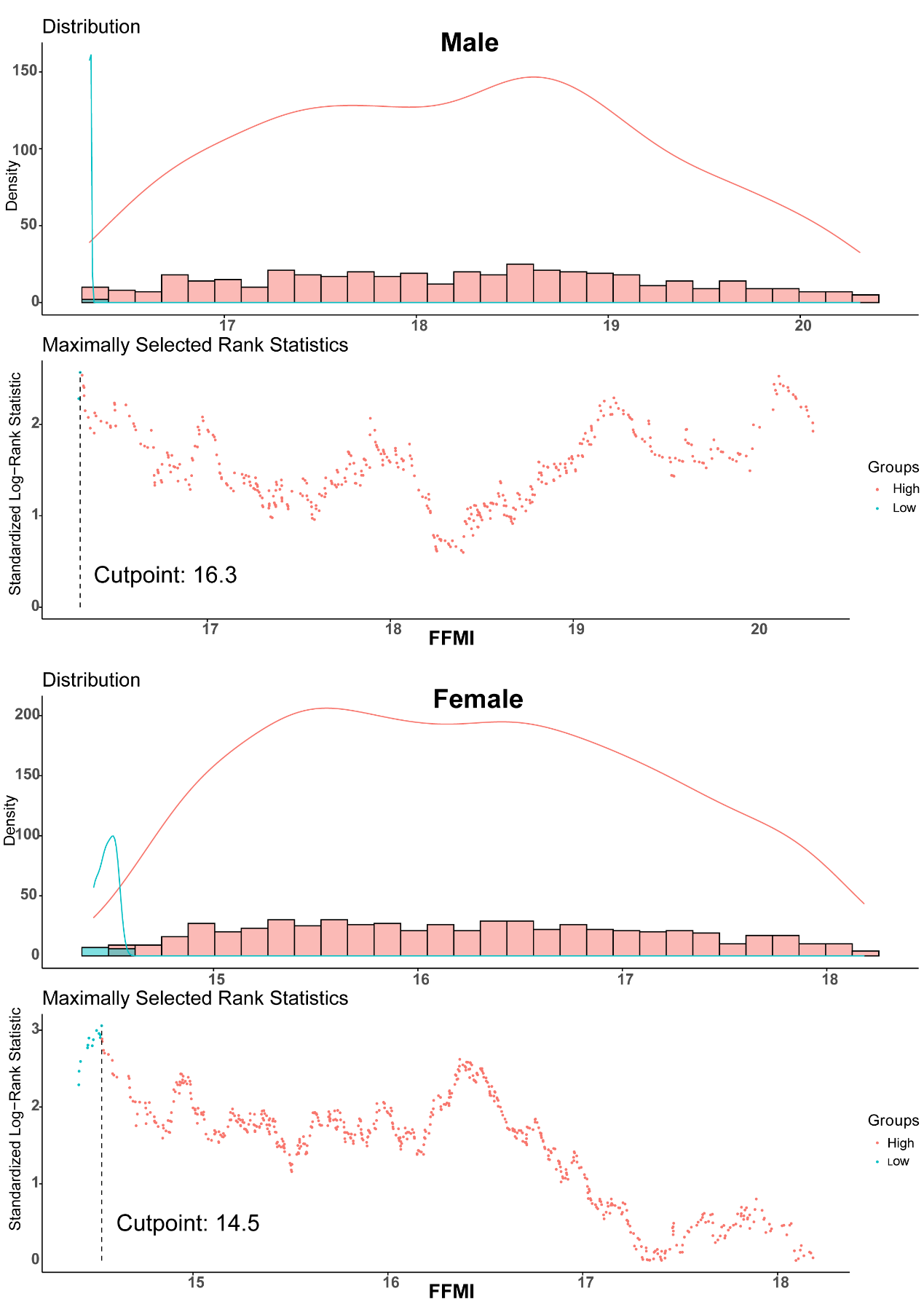


**Figure S3** Sensitivity analyses of the relationship between low FFMI and worse prognosis. a the analysis excluding patients who died within 6 months; b the analysis after adjusting using propensity score matching.


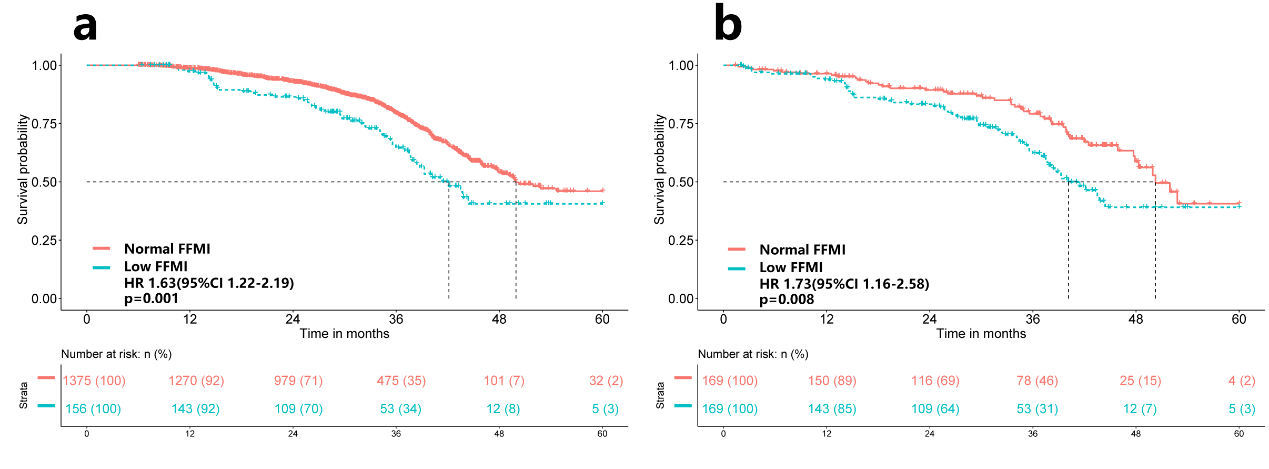


According to both analyses, low FFMI was associated with a poor prognosis after adjustment by tumor type, ECOG performance status, TNM stage, reduced food intake and radiotherapy.

**Figure S4** Kaplan-Meier survival analysis of FFMI stratified by tumor type. a OS of lung cancer subgroup; b OS of upper gastrointestinal cancer subgroup; c OS of colorectal cancer subgroup; d OS of breast cancer subgroup. OS, overall survival.


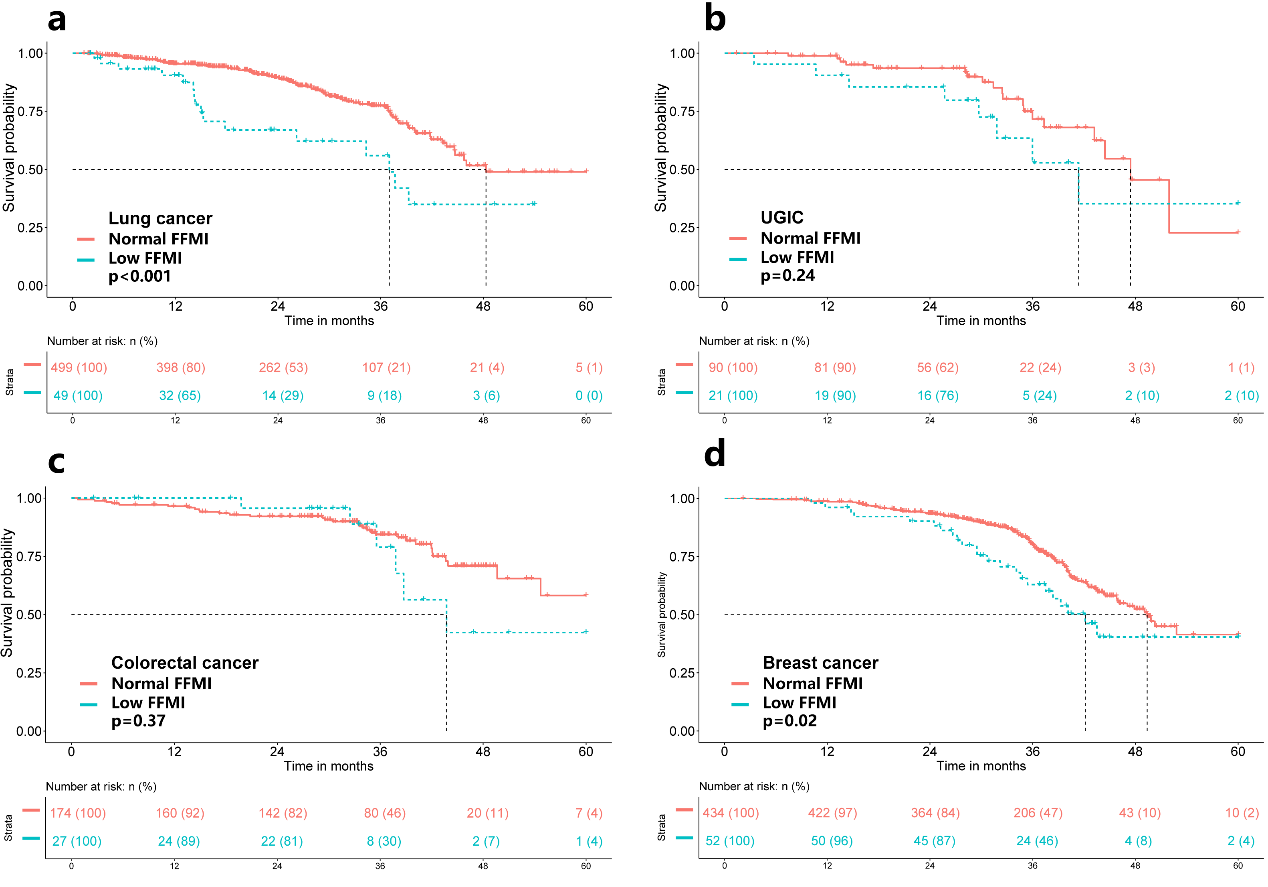


FFMI: fat free mass index; OS; overall survival; UGIC: upper gastrointestinal cancer.

**Figure S5** Kaplan-Meier survival analysis of FFMI stratified by cancer treatment. a OS of patients received surgery; b OS of patients received chemotherapy; c OS of patients received radiotherapy. OS, overall survival.


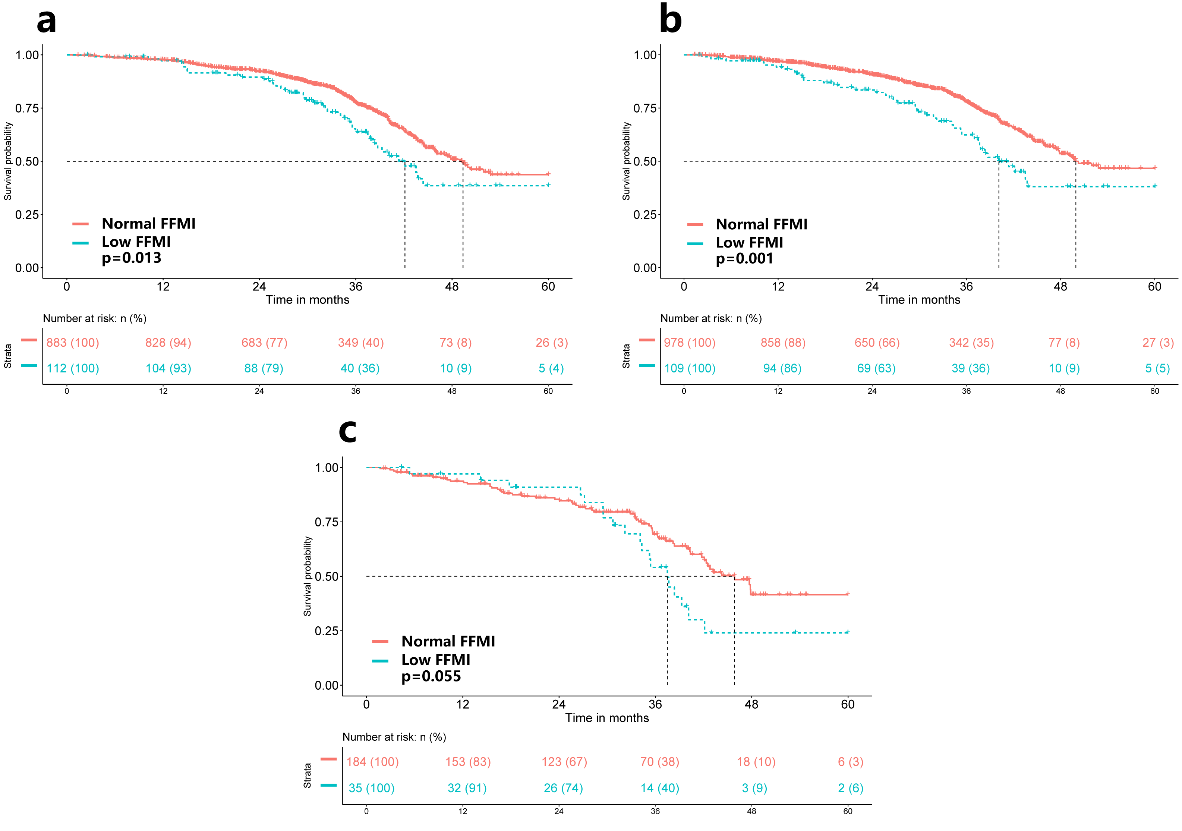


**Table S1** Clinicopathological characteristics in cancer patients with normal/high BMI after adjustment using propensity score matching

| **Characteristics** | **Normal FFMI** | **Low FFMI** | ***P*-value** |
| --- | --- | --- | --- |
|  | **n=169** | **n=169** |  |
| Age, years, n (%)* |  |  | 0.794 |
| ≤65 | 130(76.9%) | 132(78.1%) |  |
| >65 | 39(23.1%) | 37(21.9%) |  |
| Gender, n (%)* |  |  | 0.301 |
| Male | 53 (31.4%) | 62 (36.7%) |  |
| Female | 116 (68.6%) | 107 (63.3%) |  |
| ECOG performance status, n (%)* |  |  | 0.561 |
| ≤ 1 | 117 (69.2%) | 112 (66.2%) |  |
| > 1 | 52 (30.8%) | 57 (33.8%) |  |
| TNM Stages, n (%) * |  |  | 0.794 |
| I | 26 (15.4%) | 28 (16.6%) |  |
| II | 43 (25.4%) | 50 (29.6%) |  |
| III | 50 (29.6%) | 45 (26.6%) |  |
| IV | 50 (29.6%) | 46 (27.2%) |  |
| Weight-loss, n (%)* |  |  | 0.520 |
| Absent | 132 (78.1%) | 127 (75.1%) |  |
| Present | 37 (21.9%) | 42 (24.9%) |  |
| Tumor types, n (%)* |  |  | 0.284 |
| Lung cancer | 57 (33.7%) | 48 (28.4%) |  |
| UGIC | 12 (7.1%) | 21 (12.4%) |  |
| CRC | 19 (11.2%) | 27 (16.0%) |  |
| Breast cancer | 58 (34.3%) | 52 (30.8%) |  |
| Other | 23(13.6%) | 21 (12.4%) |  |
| Radiotherapy, n (%)* |  |  | 0.509 |
| Yes | 39(23.1%) | 34(20.1%) |  |
| No | 130(76.9%) | 135(79.9%) |  |
| Reduced food intake, n (%)* |  |  | 0.641 |
| Absent | 117(69.2%) | 113(66.9%) |  |
| Present | 52(30.8%) | 56(33.1%) |  |
| NLR, n (%)* |  |  | 0.514 |
| Low (≤2.46) | 89(52.7%) | 83(49.1%) |  |
| High (>2.46) | 80(47.3%) | 86(50.9%) |  |

*These variables were included in the propensity score matching analysis. Abbreviations: ECOG: Eastern Cooperative Oncology Group; UGIC: upper gastrointestinal cancer; CRC: colorectal cancer; NLR: neutrophil-to-lymphocyte ratio.

**Table S2** Univariate and multivariate analysis of FFMI combined with weight loss for OS in cancer patients with normal/high BMI

| **Group** | **Number**  **(cases/total)** | **Univariate Analysis** | | | **Multivariate Analysis** | | |
| --- | --- | --- | --- | --- | --- | --- | --- |
|  |  | **Hazard Ratio** | **95% CI** | ***P-value*** | **Hazard Ratio** | **95% CI** | ***P-value*** |
| Normal FFMI-No WL | 1242/1602 | Reference |  |  |  | Reference |  |
| Normal FFMI-WL | 190/1602 | 1.20 | 0.86 to 1.66 | 0.280 | 0.98 | 0.70 to 1.38 | 0.926 |
| Low FFMI-No WL | 127/1602 | 1.57 | 1.14 to 2.14 | 0.005 | 1.45 | 1.05 to 1.99 | 0.022 |
| Low FFMI-WL | 43/1602 | 3.18 | 1.89 to 5.36 | <0.001 | 3.53 | 2.04 to 6.11 | <0.001 |

*Adjusted for age, gender, tumor type, Eastern Cooperative Oncology Group performance status, TNM stage, neutrophil-to-lymphocyte ratio, weight loss, reduced food intake and radiotherapy.
